# Chronic Resistance Training Effects on Serum Adipokines in Type 2 Diabetes Mellitus: A Systematic Review

**DOI:** 10.3390/healthcare11040594

**Published:** 2023-02-16

**Authors:** Pablo Jiménez-Martínez, Rodrigo Ramirez-Campillo, Carlos Alix-Fages, Javier Gene-Morales, Amador García-Ramos, Juan C. Colado

**Affiliations:** 1 Research Group in Prevention and Health in Exercise and Sport (PHES), Department of Physical Education, University of Valencia, 46010 Valencia, Spain; 2 Exercise and Rehabilitation Sciences Institute, School of Physical Therapy, Faculty of Rehabilitation Sciences, Universidad Andres Bello, Santiago 7591538, Chile; 3 Applied Biomechanics and Sport Technology Research Group, Department of Physical Education, Autonomous University of Madrid, 28049 Madrid, Spain; 4 Research Institute on Traffic and Road Safety, University of Valencia, 46010 Valencia, Spain; 5 Department of Physical Education and Sport, Faculty of Sport Sciences, University of Granada, 18500 Granada, Spain; 6 Department of Sports Sciences and Physical Conditioning, Universidad Católica de la Santísima Concepción, Concepción 4090541, Chile

**Keywords:** human physical conditioning, muscle strength, metabolic diseases, glycemia, cardiometabolic risk, impaired metabolism

## Abstract

(1) Background: Non-communicable diseases (NCD) are an important concern for public health because of their high rates of morbidity and mortality. A prevalent lifestyle-linked NCD is type 2 diabetes mellitus (T2D). Recently, molecular biomarkers secreted by adipocytes, called adipokines, have been linked with T2D and muscle function disturbances. However, the effects of resistance training (RT) interventions on adipokine levels in patients with T2D have not been systematically studied. (2) Methods: The PRISMA guidelines were followed. Searches for the studies were performed in the PubMed/MEDLINE and Web of Science electronic databases. Eligibility criteria included: (i) participants with T2D; (ii) RT interventions; (iii) randomized controlled trials; and (iv) measurement of serum adipokines. The PEDro scale was used to assess the methodological quality of the selected studies. Significant differences (*p* ≤ 0.05) and effect size were screened for each variable. (3) Results: Of the initial 2166 records, database search extraction yielded 14 studies to be included. The methodological quality of the included data was high (median PEDro score of 6.5). Analyzed adipokines in the included studies were leptin, adiponectin, visfatin, apelin, resistin, retinol-binding protein 4 (RBP4), vaspin, chemerin, and omentin. RT interventions (6–52 weeks; minimal effective duration >12 weeks) exert a meaningful effect on serum adipokine, (e.g., leptin) levels in T2D patients. (4) Conclusions: RT may be an alternative, but not an optimal, option in adipokine disruptions in T2D. Combined (i.e., aerobic and RT) long-term training may be considered the optimal intervention for treating adipokine level disturbances.

## 1. Introduction

Type 2 diabetes mellitus (T2D) entails an alteration in the insulin and glucose metabolism (i.e., hyperglycemia) [1]. It is the most prevalent (i.e., 85%) type of diabetes [2]. Nearly 6.3% of the world population (462 million) suffer from TD2, with an estimated increase of up to 600 million by 2025 [3]. Health costs are 3.2 times greater in T2D patients compared with healthy subjects, and up to 9.4 times greater among T2D patients with comorbidities [2]. TD2 may lead toward retinopathy, renal malfunction, peripheral neuropathy, micro–macro vascular complications, and comorbidities that increase death risk 2–3 fold [3]. Indeed, ~89% of patients with TD2 have ≥2 comorbidities (e.g., hypertension, being overweight, dyslipidemia) [4]; some of them (e.g., being overweight) associated with mitochondrial dysfunction, hormonal dysregulation, and reduced motor function [5]. Additionally, T2D relates to skeletal muscle reduced glucose uptake, insulin resistance, chronic lipid toxicity, myosin glycation, alteration in intracellular and sarcoplasmic adenosine ATP, reduction in nerve cell axons [5], increased dynapenia risk [5], reduced neuromuscular function, and quality of life [6,7,8].

For early detection of the aforementioned cardio-metabolic risk factors associated with T2D, adipokines have become increasingly used in recent years. Adipokines (i.e., cytokines secreted by adipose tissue) reflect adipose tissue endocrine function as it relates to metabolic homeostasis [9,10] and, compared with traditional risk markers such as glycaemia or insulin, may provide better risk assessment in T2D in relation to cardiovascular health, oxidative stress, energy systems behavior, visceral fat, chronic inflammation, and comorbidities [10]. Common adipokines include leptin, adiponectin, visfatin, apelin, resistin, retinol-binding protein 4 (RBP4), vaspin, chemerin, and omentin [8].

Resistance training (RT) might reduce chronic low-grade inflammation, thus improving glucose homeostasis and insulin sensitivity in T2D [11,12]. Guidelines from exercise-related international organizations suggest moderate–vigorous RT loads in T2D: involving large muscle groups, ≥2 sessions per week, 8–10 exercises per session, 2–4 sets per exercise, 8–10 repetitions per set, and 1–2 min of inter-set recovery [13]. Health-related organizations, such as the American Diabetes Association, have also published RT guidelines, although with different programming configurations [11]. Aside from the lack of consensus regarding optimal RT programming in T2D, contrasting results have been reported in the literature regarding the effects of RT on adipokines in T2D [14,15,16,17]. Such conflicting results are in line with different RT protocols (e.g., moderate vs. high intensity; 6 vs. 48 weeks) [15,16,17,18,19]. Further, the studies that have assessed the effects of RT on adipokines in T2D usually involve a reduced sample size of 10–15 participants [18,19,20], which is a serious problem in this field [21]. At present, it is unclear how to optimize RT prescription for T2D with respect to adipokine improvement. Aminilari et al. (2017) [14] compared omentin responses to RT under a 3 × 8 at 50% RM protocol, 20–25 min of aerobic training at 50% of the maximum heart rate (HRmax), a combination of both interventions, or a passive control group. On the other hand, Kim et al. (2014) [17] evaluated the impact of a resistance training circuit of unspecified exercises at 3 × 20/50% RM combined with 30 min of aerobic training at 50–70% VO2max in comparison with a passive control intervention (i.e., without an exercise program) on chemerin, adiponectin and retinol-binding protein 4 (RBP4) levels.

For this purpose, a systematic review may provide an overview of the currently available literature, favoring an adequate perspective for the advancement in the field. Furthermore, these results will be useful to optimize the prescription of RT (e.g., intensity, volume) in this clinical population. Therefore, the primary aim of this systematic review was to determine the effects of RT on adipokine levels in T2D. The research question was: how does RT affect adipokines in type 2 diabetes mellitus compared with a control group?

## 2. Materials and Methods

This systematic review was conducted according to previous guidelines [22]. The protocol was pre-registered as a Master of Science degree project at the University of Valencia and approved by a review board of experts in the field. Meta-analysis could not be performed due to insufficient data on each adipokine. The protocol is available upon reasonable request.

### 2.1. Eligibility Criteria

Following a population, intervention, comparison, and outcome (PICO) approach, studies were included when the following criteria were satisfied: (a) involved adults or older adults (≥18 years) with T2D in the intervention group; (b) participants were enrolled in a RT program (e.g., free weights; elastic bands; body mass-based); (c) RT was compared with a contrast group (e.g., control; alternative training method) in a randomized-controlled design; and (d) serum adipokines were measured before and after the interventions.

### 2.2. Information Sources

Between 7 January 2023 and 2 February 2023, the search for studies was conducted on the following databases: Web of Science (WOS) Core Collection, Cumulative Index to Nursing and Allied Health (CINAHL), Cochrane Library, EMBASE, Scopus, SPORTDiscus, and PubMed (Medline), without the application of filters. A manual search was also performed in the reference list of each eligible study. A search for errata and retractions was carried out on the eligible studies. Pre-registered documents or complementary data were not included.

### 2.3. Search Strategy

Two authors (PJM and CAF) performed independent searches under the supervision of a third author (JCC). The search was not limited to the date of publication or language. The general search strategy made use of free text terms, MeSH terms, and the Boolean operators AND/OR, with three lines of code being implemented in “all fields” (PubMed) and “theme” (WOS): (resistance training OR strength training OR strength exercise OR resistance exercise OR weight training OR hypertrophy training OR weightlifting) AND (adipokine OR adipokines OR adipocytokines OR leptin OR adiponectin OR visfatin OR apelin OR omentin OR resistin OR retinol-binding protein 4 OR adipsin OR vaspin OR chemerin) AND (diabetes OR diabetic OR diabetes Mellitus OR type 2 diabetes).

### 2.4. Selection Process

PJM and CAF independently screened each record and each report retrieved. In the case of disagreement between the two authors, JCC provided arbitrage until consensus was achieved. Automated removal of duplicates was performed using EndNoteWeb (Clarivate^TM^), but further manual removal of duplicates was required.

### 2.5. Data Extraction Process

PJM and CAF independently collected data from reports. In the case of disagreement between the two authors, JCC provided arbitrage until consensus was achieved. Where relevant data was missing and/or additional details were required, the authors of the studies were contacted (one time), and the required information was solicited. If no response was obtained, the study was excluded. However, where data were displayed in a figure [15,16,18,19], validated (*r* = 0.99, *p* < 0.001) software (WebPlotDigitizer; https://apps.automeris.io/wpd/) was used to derive the relevant numerical data [23]. Access to the software date was 10 January 2023. The collected data was allocated in a Microsoft Excel sheet template elaborated a priori.

### 2.6. Data Collection

Final collected data included: authors and year of publication; number of participants and sex; mean age and standard deviation or range; exercise modality; intervention duration (weeks); training frequency (sessions per week); and exercise protocol, including volume and intensity (e.g., RM percentage (% 1RM)). Outcome (i.e., adipokines) mean values and standard deviations (e.g., ng/mL; μg/mL) were reported pre-test and post-test. Significant difference (*p* ≤ 0.05) was declared for each outcome according to the original research values. Effect size (ES) for mean differences of groups within a pre-post design was calculated for each outcome according to previous research [24]. ES was calculated using the following scale: negligible (<0.20), small (0.20–0.49), moderate (0.50–0.79), and large (≥0.80) [24]. Some of the selected studies [19,20] included active control groups in comparison with the experimental RT group. For those studies, data related to exercise type (e.g., running), volume (minutes), and intensity (e.g., HRmax; reserve heart rate (HRR); maximal oxygen uptake (VO2max)) were also considered. Moreover, in one of the studies, the comparison was performed between diabetic and non-diabetic patients, both enrolled in a RT protocol [18]. All studies included a T2D-diagnosed intervention group.

### 2.7. Methodological Quality of the Included Studies

The Physiotherapy Evidence Database (PEDro) scale was used to assess the methodological quality of the included studies, which were rated from 0 (lowest quality) to 10 (highest quality). The validity and reliability of the PEDro scale have been established previously [25,26]. Additionally, its agreement with other scales (e.g., Cochrane risk of bias tool) has been reported [27]. Moreover, the PEDro scale is the checklist most frequently used in RT-related literature (e.g., plyometric training) [28]. According to cut-off scores, the methodological quality was rated as ‘poor’ (<4), ‘fair’ (4–5), ‘good’ (6–8) and ‘excellent’ (9–10) in some sub-fields; however, it is not possible to satisfy all scale items in some areas of physiotherapy practice [29]. Therefore, as outlined in previous systematic reviews, the methodological quality of RT studies was interpreted using the following convention [28]: ≤3 points was considered poor quality, 4–5 points was considered moderate quality, and 6–10 points was considered high quality. If trials were already rated and listed in the PEDro database, the respective scores were adopted. The methodological quality for each included study was assessed independently by two authors (PJM and CAF), and any discrepancies between them were resolved via consensus with a third author (RRC).

## 3. Results

### 3.1. Data Selection

**Figure 1:**
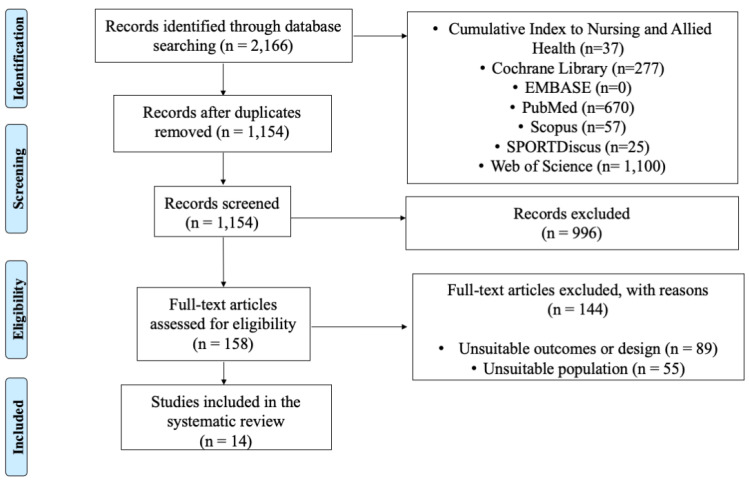
Flow chart illustrating the study selection process.

Database searches allowed the discovery of 2166 documents (see Figure 1). After duplicates were removed and records screened, only 158 full-text articles were potentially eligible. Finally, 14 studies were included in the systematic review. Data selection is described in Figure 1.

### 3.2. Studies’ Characteristics

The studies’ characteristics are presented in the Table 1.

The total aggregated participants of this systematic review were 619, of which 413 were females and 206 were males. Age ranged between 45 [18] and 70 years [16]. Most of the included studies used external loads such as elastic bands, resistance training machines, or free weights [16,30,31]. Study durations ranged from 6 [18] to 52 weeks [15] and participant numbers oscillated between 15 [19] and 90 [16]. Only in one study did participants not present as overweight (i.e., body mass index ≥ 25 kg/m^−2^). No non-chronic comorbidities (e.g., cardiovascular disease) were reported in any of the selected studies. T2D onset was at least two years in all the chosen research. Of the 14 selected studies: in 7 studies, participants were enrolled on aerobic training, RT, aerobic + RT, or a control group; in 2 studies, participants performed RT or a passive control; 2 studies compared RT with a passive control; 1 research selected diabetic vs non-diabetic intervention [18]; and 2 studies included RT and aerobic exercise.

### 3.3. Methodological Quality of Included Studies

According to the modified PEDro scale, the included studies reached a median high-quality score of 6.5 (Table 2). Twelve of the studies were considered as “high quality” (6–8 points) and two were rated as “medium quality” (4–5 points). None of the included studies was classified as “poor quality” (<3 points) (Table 2).

### 3.4. Results Synthesis

Of the 14 selected studies, adiponectin was measured in 9, leptin in 5, RBP4 in 4 and resistin and visfatin in 3. On the other hand, vaspin, omentin, chemerin and apelin only were identified in one study for each.

The studies’ results are presented as *p*-value and effect size in Table 3.

**Figure 2:**
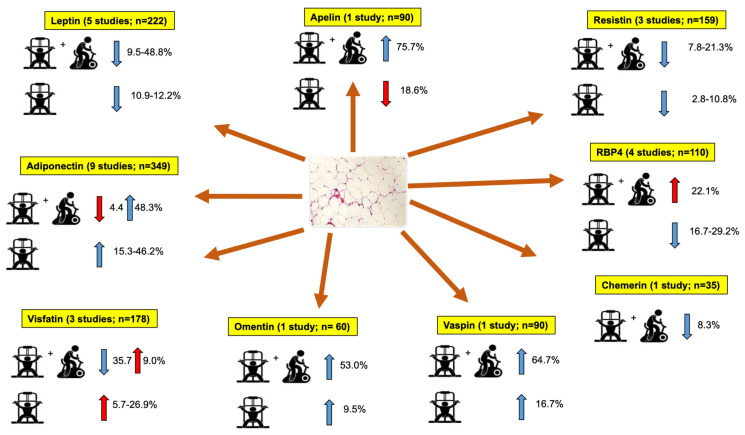
Effects of resistance training and combined resistance and aerobic training on adipokines. Yellow box: denotes the adipokine, the number of studies that analyzed the adipokine, and the total number of participants in the studies; Red arrow: result favored the control group; Blue arrow: result favored the training group; Range values: represent the results from two or more studies; RBP4: Retinol-binding protein 4.

Figure 2 graphically depicts a summary of the effects of RT on adipokines in type 2 diabetes mellitus.

#### 3.4.1. Leptin

Regarding leptin levels, Ku et al. [33] observed a 12.2% reduction after RT, 37.8% after aerobic training, and 0.9% after control conditions in a 12-week intervention. Kanaley et al. [18] reported a 10.9% reduction after RT in T2D participants compared with a 4.4% increase in healthy participants after 6 weeks of interventions. Studies that combined RT and aerobic training [15,33,34] observed a 9.5% to 48.8% decrease after combined training, a 9.7% to 20.2% decrease after aerobic training, and changes from −11.6% to +6.8% in control groups after 16–52 weeks.

#### 3.4.2. Adiponectin

Annibalini et al. [30] reported a 4.3% reduction in adiponectin levels after RT combined with aerobic exercise and a 3.4% reduction in the passive control group in a 16-week intervention. In a 52-week study, Balducci et al. [15] observed a 48.3% increase after combined exercise, a 1.9% to 29.7% reduction after aerobic training, and a negligible 0.1% increase after the control conditions. Another study [17] found a 16.1% increase after combined training and a 10.5% reduction after the control condition in a −12-week protocol. Five studies [20,30,31,34,36] observed a 15.3% to 46.2% increase after RT, with −26.0% to 41.2% variations in the passive control groups, and a 35.3% increase after aerobic training in 12–48 weeks of interventions. In a 12-week design [33], a 15.3% increase was reported after RT, a 10.0% increase after combined training, and a 26.0% and 39.4% decrease after aerobic training and stretching control conditions, respectively. After 12 weeks, one intervention [19] reported a 46.0% increase in the combined group and 35.3% in the active aerobic control group.

#### 3.4.3. Visfatin

In a 12-week intervention, one study [32] observed 26.9%, 17.2%, 9.0%, and 29.8% increases after RT, aerobic training, combined training, and stretching control conditions, respectively. Another intervention [16] reported a 5.7% increase after RT, a 32.3% reduction after aerobic training, and a 35.7% decrement after combined training, with a negligible reduction (1.2%) after leisure aerobic control condition in a 24-week design.

#### 3.4.4. Apelin

After 24 weeks, an 18.6% reduction in apelin levels was reported in the RT group, and a 67.1%, 75.7%, and 4.4% increase after aerobic training, combined training, and leisure aerobic control conditions, respectively [16].

#### 3.4.5. Resistin

Balducci et al. [15] found 21.3%, 4.5% to 14.6%, and 2.7% reductions in resistin levels after combined training, different aerobic training modalities, and passive control conditions, respectively, in a 52-week intervention. Another study [32] reported 10.8%, 2.0%, 7.8%, and 2.7% reductions after RT, aerobic training, combined training, and stretching control conditions, respectively, in a 12-week experiment. After 48 weeks, Miller et al. [36] observed 2.8% and 18.4% reductions in the RT group and control conditions, respectively.

#### 3.4.6. Retinol-Binding Protein 4 (RBP4)

In a 16-week experiment, Annibalini et al. [30] observed 28.1% and 3.6% reductions after RT and the control condition, respectively. After 12 weeks, Kang et al. [19] reported 29.2% and 11.0% reductions after RT and the active aerobic control conditions, respectively. After 12 weeks, Kim et al. [17] found a 22.1% and 3.5% increase in combined training and the control conditions, respectively. Ku et al. [33] observed 16.7% and 2.6% reductions after RT and aerobic training, and a 1.3% increase in the control condition, respectively, in a 12-week protocol.

#### 3.4.7. Vaspin

After 24 weeks, Kadoglou et al. [16] reported 16.7%, 44.4%, 64.7%, and 7.4% increases in vaspin levels after RT, aerobic training, combined training, and control conditions (i.e., leisure aerobic activities), respectively.

#### 3.4.8. Chemerin

Kim et al. observed an 8.3% decrement in chemerin levels after combined training and an 8.1% increase after the control condition in a 12-week experiment [17].

#### 3.4.9. Omentin

After 12 weeks of intervention, Aminilari et al. [14] reported a 5.1%, 9.5%, and 53.1% increase in omentin levels after aerobic training, RT, and combined training, respectively, and an 11.4% reduction after the control condition.

## 4. Discussion

The primary aim of this systematic review was to determine the effects of RT on adipokines in T2D. The main findings indicate that leptin, apelin, and vaspin respond better to resistance training combined with aerobic training compared with any other exercise intervention. It was also found that resistance training exerts a better response on chemerin, resistin, adiponectin, RBP4 and omentin in comparison with aerobic training or passive control conditions. Inconclusive results were reported for visfatin. As a result, considering that adipokines are predictive biomarkers for metabolic disorders and comorbidities [8,10], the current results offer valuable insights regarding the therapeutic role of RT as a non-pharmacological treatment for T2D patients. Thus, the effects of each exercise program on each particular adipokine will be discussed hereunder.

Leptin is a key hormone in the management of hyperphagia, systemic inflammation, overweight conditions, and insulin resistance [37]. High leptin levels and leptin resistance have been linked to pathological dysfunctions and metabolic syndromes [36]. Our findings suggest that RT interventions combined with aerobic exercise [15,32,33] induced greater reductions in leptin levels (i.e., up to 48.8%) in comparison with active control, aerobic, or resistance exercise alone. In this regard, interventions involving RT alone showed leptin reductions of up to 12.2% [18,31]. The leptin reduction following RT may be related to reductions in insulin values, body mass, and fat mass [38]. Of note, greater relative [15] and absolute [18] leptin reductions were noted among participants with higher pre-intervention leptin levels, as previously suggested [39]. Overall, RT, when combined with aerobic training, seems to offer the greatest potential benefits on leptin levels.

Concerning adiponectin, its physiological functions are presented in visceral adipose tissue, inducing liver fatty acid oxidation and reducing hepatic lipogenesis, and peripheral tissues where glycemia control and insulin resistance are influenced by this biomarker [8]. In our data, RT, when compared with combined training, induced a similar increase in adiponectin levels (i.e., up to 46.2–48.3%) [15,17,19,30,31,32]. Moreover, RT, compared with aerobic training or passive controls, induced greater adiponectin rises (up to 35.3%) [19,20,37,38]. The reported rise in adiponectin levels may be beneficial due to reductions in lipoinflammation and oxidative stress, an increase in Adipo 1–2 receptors and PPARα activity, and a reduction in visceral metabolic risk [40,41,42,43]. Overall, RT alone seems to raise adiponectin levels as much as other types of exercise, which may be valuable in the management of metabolic dysfunctions.

Another important adipokine in metabolic disease management is visfatin [44]. This adipokine exerts its physiological functions through the control of insulin levels, inflammation and reactive oxygen species regulation [45]. Moreover, high levels of visfatin have been linked to insulin resistance and obesity [45]. Our findings suggest that combined training reduced visfatin up to 35.7%, with a negligible impact after RT alone (i.e., 5.7–26.9% increase) [16,30,45]. These results may be connected to the impact of RT on short-term increases in reactive oxygen species production and inflammation, and long-term antioxidant adaptive responses [46,47]. Therefore, RT may acutely alter visfatin levels; however, relatively longer interventions may be needed to clarify more precise implications of RT on visfatin levels in this population.

With regard to apelin, previous research has associated low levels with an increase in metabolic risk [48,49,50]. An increase in apelin (i.e., raised to levels between 1.63–3.52 ng/mL) [48,49] may lead to improved vasodilation, heart contractility, GLUT 4 activity and energy metabolism in overweight, hyperlipidemia and diabetes patients [50,51]. Similar to visfatin, combined training raised apelin levels (i.e., 75.7% increase), with no favorable impact after RT alone (i.e., 18.6% reduction) [16]. Such improvements of combined training on apelin levels might be mediated by positive changes in body composition and stimulation of glucose turnover [50,51,52].

The adipokine resistin has been linked to T2D, and has thus been suggested in recent years as a preclinical marker of insulin resistance [53]. To date, interventions involving RT alone have observed reductions in resistin levels after RT (i.e., up to 10.8%) and larger changes after combined training (i.e., up to 21.3%) [15,30,38]. Overall, although combined training may exert the greatest benefits on resistin levels, RT alone may be an alternative, promoting anti-inflammatory pathways, reducing insulin resistance and improving glucose tolerance through resistin levels reductions [54].

RBP4 plays a key role in glucose homeostasis and GLUT transporter efficiency, linking altered metabolic states to diabetes risk [55]. RT exerts greater reductions (i.e., up to 29,2%) than aerobic training (i.e., up to 11.0%) and combined training (i.e., increments up to 22.1%) [17,19,31,35]. As mentioned, the mechanism underlying RT benefits may be linked to improvements in insulin sensitivity and the activity of peripheral GLUT transporters. However, this adipokine is manifested in different forms in humans [56], which may be contextualized in future research. Collectively, RT exhibits the greatest improvements in this biomarker, helping to reduce hyperglycemia and glycosylated hemoglobin, and improving peripheral insulin sensitivity.

Concerning vaspin, this adipokine exerts functions related to the control of systemic insulin resistance, reactive oxygen species (ROS), hyperlipidemia, and inflammation due to its visceral origin [46,57]. Only one study has already evaluated the impact of RT on its levels. A 4-fold rise in vaspin levels in the combined training group (i.e., up to 64.7%) and a 3-fold increase in the aerobic training group (i.e., up to 44.4%) in comparison with RT (i.e., up to 16.7%) was found [16]. Our findings suggest that RT may be not optimal for improving vaspin levels because of insufficient antioxidant and anti-inflammatory short-term activity [16]. Overall, the greatest results were found when aerobic training was included alone or in combination with resistance training, which may be helpful in the management of oxidative stress and insulin resistance [46,58].

Another important adipokine in diabetes care is chemerin. High levels of chemerin are associated with insulin over-production, hypertension, high glycosylated hemoglobin levels and endothelial damage [59]. In relation to chemerin, combined training indicates greater reductions (i.e., up to 8.3%) in comparison with control conditions (i.e., chemerin levels increase up to 8.1%) in the only selected study [17]. Overall, combined training exhibits the greatest benefits in the management of chemerin levels due to improvements in insulin resistance. Therefore, the inclusion of RT in a combined protocol may exert the largest effects on chemerin levels [56,59].

Finally, the last adipokine collected was omentin. This biomarker plays its physiological roles through a visceral adipose tissue insulin-sensitivity mechanism [58]. Moreover, RT may exert its benefits through reductions in visceral adipose tissue infiltration and upregulating insulin sensitivity [58]. Our data found a rising effect of RT (i.e., up to 9.5%) and combined training (i.e., up to 53.0%) compared with aerobic training (i.e., reductions up to 5.1%) [14]. Thus, RT alone or embedded in an aerobic training program may be a potential therapeutic tool for this biomarker. These effects may positively influence cardiometabolic risk and visceral insulin resistance in T2D patients, which may lead to important reductions in the main comorbidities of this population [58,60].

Collectively, although our findings provide promising data, current literature in this field is not conclusive regarding RT effects on adipokines in T2D patients. Despite some moderate methodological quality interventions, the median PEDro score is considered high-quality (i.e., 6.5). However, selected studies exhibit heterogeneity in training protocol modalities and variables (e.g., different materials used, volume, percentage of resistance, effort type, etc.). Furthermore, a meta-analysis could not be performed due to the small number of studies for each adipokine. Thus, future research may focus on more accurate monitoring of training variables and intra-intervention standardization. Hence, designing optimal protocols is important because RT adaptations are specific to the efforts performed. Another limitation of this review is related to unexpressed direct data in some of the selected studies, forcing us to obtain data from graphics through computer software (WebPlotDigitizer). However, although some limitations are noted, a summary of the training protocols analyzed that reported greater improvements for specific adipokines is shown in Table 4.

## 5. Conclusions

Resistance training alone or combined with aerobic exercise exerts a positive impact on serum adipokines. However, distinct responses for each biomarker were reported. Greater effects were noted after prolonged physical exercise interventions (>12-week interventions). Compared with passive controls, resistance training elicits improvements in most serum adipokines. However, the magnitude, strength, and direction of these relations are specific for each molecule. Valuable information for healthcare professionals and sports scientists is related to an adequate and precise exercise “dosage”. Practical applications for each molecule are depicted in Table 4, which could be helpful in the design of evidenced-based exercise programs.

## Figures and Tables

**Table 1:** Exercise protocols of the included studies.

Study ^¶^	Sample *n* (Sex), Age, Time in DM2, Comorbidities	Exercise Type	Duration (W)	Frequency (W)	Exercise Protocol
Aminilari et al., 2017 [14]	45 (F) 45–60 years >2 years Without cardiovascular or chronic conditions	RT AET RT + AET C	12	3	RT: 6 exercises, 3 × 8/50–55% RM AET: 20–25 min/50–55% HRmax RT + AET: 6 exercises, 1–2 × 8/50–55% RM + 10–12 min/50–55% HRmax C
Annibalini et al., 2017 [30]	16 (M) 55–70 years 7.8–10.1 Without cardiovascular diseases or medication	RT + AET C	16	3	RT + AET: 4 exercises, 2–4 × 12–20/40–60% RM + 30–60 min/40–65% HRR C
Balducci et al., 2010 [15]	82 (34 F, 48 M) 50–70 years 7.8–10.1 Without cardiovascular diseases or medication	AET 1 AET 2 RT + AET C	52	2	AET 1: unspecified AET 2: 70–80% VO2max, 60 min RT + AET: 70–80% VO2max, 40 min + 4 exercises/80% 1RM 20 min C
Brooks et al., 2007 [31]	62 (22 F, 40 M) >55 years 8–11 years Without cardiovascular or chronic conditions	RT C	16	3	RT: 5 exercises, 3 × 8/60–80% RM, 35 min C
Jorge et al., 2011 [32]	48 (30 F, 18 M) 53.9 ± 9.9 years 5–7 years Without cardiovascular complications	RT AET RT + AET C	12	3	RT: Circuit of 7 exercises, 60 min. Intensity unspecified AET: Lactic threshold HR, 60 min RT + AET: Circuit of 7 exercises, 30 min Intensity unspecified + Lactic threshold HR, 30 min C: Stretching, 60 min
Kadoglou et al., 2013 [16]	90 (65 F, 25 M) 56–70 years 5–7 years Without cardiovascular, orthopedic, immune or cytokine-derived disruptions	RT AET RT + AET C	24	4	RT: 8 exercises, 2–3 × 8–10/60–80% RM, 60 min AET: 60–75% HRmax, 60 min RT + AET: 1 session of RT, 1 session of AET, 2 sessions RT + AET C: Leisure time activities, 150 min/week
Kanaley et al., 2001 [18]	30 (15 F, 15 M) 45–55 years Non-detailed Without cardiovascular or other metabolic diseases	RT RT	6	3	Diabetics: 1 exercise per muscle group, 3 × 8–12/80% 3RM Non–diabetics: 1 exercise per muscle group, 3 × 8–12/80% 3RM
Kang et al., 2009 [19]	15 (F) 51.5 ± 2.2 years Non-detailed Without complex metabolic alterations	RT AET	12	3	RT: 3 × 12/50–55% HRR AET: 20–25 min/50–55% HRmax
Kim et al., 2014 [17]	35 (19 F, 17 M) 48.34 ± 8.4 years 7.9–10 years Without cardiovascular or chronic conditions	RT + AET C	12	3	RT + AET: Circuit with unspecified exercises, 3 × 20/50% 1RM, 40 min + 30 min/50–70% V02max C
Ku et al., 2010 [33]	44 (F) 56.4 ± 7.1 years 5.8–6.6 years Without cardiovascular or renal conditions	RT AET C	12	5	RT:10 exercises, 3 × 15–20 with elastic bands EA: 3.6–5.2 METs/30 min. C
Loimaala et al., 2009 [34]	50 (M) 58.3 ± 1.81 years Non-detailed Non-detailed	RT + AET C	24	4 (2 days RT, 2 days AET)	RT + AET: 8 exercises, 3–4 × 10–12/80% RM + 60–80% VO2max (time unspecified) C
Mehdizadeh et al., 2016 [35]	40 (F) 45–60 years 6.9–9.1 years Without acute or chronic conditions	RT AET RT + AET C	12	3	RT: 3 × 10/50–65% RM AET: 20–50 min/60–80% HRmax RT + AET: 3 × 10/50–65% RM + 20–50 min/60–80% HRmax C
Miller et al., 2017 [36]	29 (16 F, 13 M) 67.2 ± 5.2 years 7.6–8.8 years Non-detailed	RT C	48	3	RT: 3 × 8/60–85% RM, 45 min C
Sukala et al., 2012 [20]	18 (13 F, 5 M) 49.0 ± 5.0 years 2.6–3.3 years Without acute or chronic conditions	RT AET	16	3	RT: 8 exercises, 2–3 × 6–8, 40–60 min AET: 60–65% HRR, 40–60 min

Abbreviations (ordered alphabetically): AET: aerobic training; C: control; F: females; HRmax: maximum heart rate; HRR: heart rate reserve; M: males; 1RM: one repetition maximum; RT: resistance training; S: sessions; VO2max: maximal oxygen uptake; W: week. ^¶^: all studies included participants with body mass index ≥ 25.0 kg/m^−2^, except in the Kang et al. study (i.e., 23.4 kg/m^−2^).

**Table 2:** Results from the PEDro checklist.

Criteria *	Aminilari et al., 2017 [14]	Annibalini et al., 2017 [30]	Balducci et al., 2010 [15]	Brooks et al., 2007 [31]	Jorge et al., 2011 [32]	Kadoglou et al., 2013 [16]	Kanaley et al., 2001 [18]	Kang et al., 2009 [19]	Kim et al., 2014 [17]	Ku et al., 2010 [33]	Loimaala et al., 2009 [34]	Mehdizadeh et al., 2016 [35]	Miller et al., 2017 [36]	Sukala et al., 2012 [20]
1	1	1	1	1	1	1	1	1	1	1	0	1	1	1
2	1	1	1	1	1	1	1	1	1	1	1	1	1	1
3	0	0	1	0	0	1	0	0	1	0	0	1	1	1
4	1	1	1	0	1	1	0	1	1	1	1	1	0	1
5	0	0	0	0	0	0	0	0	0	0	0	0	0	0
6	0	0	1	0	0	0	0	0	0	0	0	0	0	0
7	0	0	0	0	0	0	0	1	0	0	0	0	0	0
8	1	1	0	1	1	1	1	1	1	1	1	0	0	0
9	0	1	1	1	1	0	1	0	0	1	0	0	0	0
10	1	1	1	1	1	1	1	1	1	1	1	1	1	1
11	1	1	1	1	1	1	1	1	1	1	1	1	1	1
Total	6	7	8	6	7	7	6	7	7	7	5	6	5	6

*: detailed explanation for each PEDro scale item can be found at: https://www.pedro.org.au/english/downloads/pedro-scale accessed on 13 January 2023. Criterion achieved = 1. Criterion not achieved = 0.

**Table 3:** Effects of resistance training on serum adipokines in type 2 diabetic patients.

Study ^¶^	Adipokine	Main Outcomes: Pre (ng/mL, μg/mL)	Main Outcomes: Post (ng/mL, μg/mL)	*p*-Value	Effect Size
Aminilari et al., 2017 [14]	Omentin	RT: 29.00 (±4.90) AET: 27.67 (±7.60) RT + AET: 31.90 (±4.12) C: 24.17 (±5.75)	RT: 31.76 (±5.26) AET: 29.09 (±5.78) RT + AET: 48.82 (±65.48) C: 21.41 (±6.71)	RT: 0.59 AET: 0.66 RT + AET: 0.001 * C: 0.11	RT/C: 0.99 AET/C: 0.61 RT + AET/C: 3.77
Annibalini et al., 2017 [30]	Leptin Adiponectin RBP4	RT + AET: 5.4 (±1.8) C: 5.7 (±2.7) RT + AET: 2.3 (±0.9) C: 2.9 (±1.0) RT + AET: 30.6 (±9.7) C: 27.7 (±4.0)	RT + AET: 3.6 (±1.5) C: 5.2 (±2.5) RT + AET: 2.2 (±1.0) C: 2.8 (±1.6) RT + AET: 22.0 (±4.4) C: 26.7 (±5.1)	RT + AET/C: 0.006 * RT + AET/C: 0.897 RT + AET/C: 0.003 *	RT + AET/C: −0.54 RT + AET/C: 0.01 RT + AET/C: −0.96
Balducci et al., 2010 [15]	Leptin Adiponectin Resistin	AET1: 15.81 (±4.92) AET 2: 15.74 (±4.26) RT + AET: 15.28 (±4.92) C: 17.65 (±4.58) AET 1: 19.47 (±4.92) AET 2: 15.61 (±3.10) RT + AET: 14.57 (±1.91) C: 17.13 (±2.87) AET 1: 4.89 (±0.68) AET 2: 4.66 (±0.25) RT + AET: 4.61 (±0.29) C: 4.73 (±0.53)	AET 1: 14.27 (±4.87) AET 2: 12.55 (±2.90 RT + AET: 7.81 (±1.24) C: 15.60 (±4.64) AET 1: 19.10 (±3.67) AET 2: 20.24 (±2.19 RT + AET: 21.61 (±3.58) C: 17.14 (±2.53) AET 1: 4.67 (±0.83) AET 2: 3.98 (±0.34) RT + AET: 3.63 (±0.29) C: 4.16 (±0.37)	AET 1/AET2/RT + AET/C: 0.22 AET 1/AET2/RT + AET/C: 0.10 AET 1/AET2/RT + AET/C: 0.46	AET 1/C: 0.09 AET 2/C: −0.25 RT + AET/C: −1.13 AET 1/C: −0.09 AET 2/C: 1.54 RT + AET/C: 2.87 AET 1/C: 0.57 AET 2/C: −0.26 RT + AET/C: −0.93
Brooks et al., 2007 [31]	Adiponectin	RT: 5.1 (±1.32) C: 8.3 (±1.12)	RT: 6.6 (±1.35) C: 6.7 (±1.15)	RT/C: <0.001 *	RT/C: 2.61
Jorge et al., 2011 [32]	Resistin Visfatin Adiponectin	RT: 8.54 (±1.46) AET: 7.34 (±1.36) RT + AET: 8.21 (±3.13) C: 8.24 (±1.66) RT: 112.11 (±42.85) AET: 112.24 (±45.83) RT + AET: 116.19 (±75.41) C: 103.57 (±55.06) RT: 4.45 (±4.12) AET: 5.58 (±5.73) RT + AET: 5.98 (±3.43) C: 5.07 (±5.50)	RT: 7.62 (±1.68) AET: 7.19 (±1.08) RT + AET: 7.57 (±2.89) C: 8.02 (±1.43) RT: 142.25 (±51.04) AET: 131.54 (±58.38) RT + AET: 127.46 (±45.22) C: 134.12 (±72.06) RT: 5.13 (±4.30) AET: 3.38 (±2.22) RT + AET: 6.58 (±5.44) C: 3.75 (±2.93)	RT: >0.05 AET: >0.05 RT + AET: >0.05 C: >0.05 RT: <0.05 * AET: <0.05 * RT + AET: <0.05 * C: <0.05 * RT: >0.05 AET: >0.05 RT + AET: >0.05 C: >0.05	RT/C: −0.43 AET/C: 0.045 RT + AET/C: −0.89 RT/C: −0.003 AET/C: −021 RT + AET/C: −0.28 RT/C: 0.39 AET/C: −0.15 RT + AET/C: −0.18
Kadoglou et al., 2013 [16]	Vaspin Apelin Visfatin	RT: 0.96 (±0.31) AET: 1.17 (±0.32) RT + AET: 0.99 (±0.28) C: 1.08 (±0.31) RT: 0.59 (±0.19) AET: 0.76 (±0.21) RT + AET: 0.74 (±0.21) C: 0.68 (±0.19) RT: 30.98 (±8.42) AET: 34.92 (±7.89) RT + AET: 35.64 (±8.24) C: 30.08 (±9.14)	RT: 1.12 (±0.39) AET: 1.69 (±1.08) RT + AET: 1.63 (±0.43) C: 1.16 (±0.38) RT: 0.48 (±0.29) AET: 1.27 (±0.40) RT + AET: 1.30 (±0.32) C: 0.71 (±0.31) RT: 32.76 (±8.97) AET: 23.64 (±9.11) RT + AET: 22.92 (±5.44) C: 29.73 (±9.49)	AET/RT + AET: <0.001 * AET/RT + AET: 0.260 AET/RT + AET: <0.001*	AET/RT + AET: 0.68 AET/RT + AET: −0.23 AET/RT + AET: 0.18
Kanaley et al., 2001 [18]	Leptin	Diabetics: 41.4 (±8.9) Non-diabetics: 11.4 (±3.0)	Diabetics: 36.9 (±8.80) Non-diabetics: 11.9 (±8.8)	Diabetics: <0.05 *	Diabetics/Non-diabetics: −0.77
Kang et al., 2009 [19]	RBP4 Adiponectin	RT: 49.26 (±8.30) AET: 35.36 (±4.01) RT: 6.92 (±2.35) AET: 6.17 (±1.06)	RT: 34.87 (±2.93) AET: 31.46 (±5.36) RT: 10.11 (±2.82) AET: 8.35 (±1.44)	RT: <0.001 * AET: <0.001 * RT: <0.05 * AET: <0.05 *	RT: 34.87/AET: −1.55 RT/AET: 0.53
Kim et al., 2014 [17]	RBP4 Chemerin Adiponectin	RT + AET: 62.4 (±13.2) C: 62 (±20) RT + AET: 97.6 (±28.9) C: 103.2 (±12.7) RT + AET: 3.1 (±1.0) C: 3.8 (±1.6)	RT + AET: 76.2 (±14.6) C: 64.2 (±15.7) RT + AET: 89.5 (±24.1) C: 111.4 (±18.2) RT + AET: 3.6 (±1.3) C: 3.4 (±1.2)	RT + AET/C: >0.05 RT + AET/C: 0.021 * RT + AET/C: >0.05	RT + AET/C: 0.67 RT + AET/C: −0.70 RT + AET/C: 0.66
Ku et al., 2010 [33]	Leptin Adiponectin RBP4	RT: 8.8 (±4) AET: 9.86 (±3.06) C: 11.6 (±5.8) RT: 4.98 (±2.52) AET: 3.86 (±2) C: 4.83 (±1.99) RT: 98.5 (±28.8) AET: 87.0 (±25.4) C: 95.0 (±20.5)	RT: 7.73 (±4.05) AET: 6.13 (±4.00) C: 11.50 (±4.92) RT: 7.28 (±3.72) AET: 6.76 (±1.24) C: 6.82 (±2.39) RT: 82.1 (±27.1) AET: 84.7 (±15.3) C: 96.2 (±28.7)	RT/AET/C: >0.05 RT/AET/C: >0.05 RT/AET/C: >0.05	RT/C: −0.19 AET/C: −0.77 RT/C: 0.39 AET/C: 0.44 RT/C: −0.68 AET/C: −0.147
Loimaala et al., 2009 [34]	Leptin	RT + AET: 7.4 (±4.1) C: 7.4 (±3.8)	RT + AET: 6.7 (±3) C: 7.9 (±3)	RT + AET: 0.43 C: 0.98	RT + AET/C: −0.29
Mehdizadeh et al., 2016 [35]	Visfatin	RT: 18.67 (±1.25) AET: 25.76 (±5.18) RT + AET: 21.61 (±2.66) C: 20. 24 (±2.37)	RT: 24.94 (±4.71) AET: 15.35 (±1.35) RT + AET: 15.80 (±1.88) C: 21.90 (±2.53)	RT/AET/RT + AET/C: >0.05	RT/C: 2.33 AET/C: −2.87 RT + AET/C: −2.84
Miller et al., 2017 [36]	Resistin Adiponectin	RT: 10.54 (±5.64) C: 10.99 (±3.86) RT: 1.68 (±0.66) C: 2.67(±0.95)	RT: 10.24 (±5.34) C: 8.97 (±3.91) RT: 1.94 (±0.93) C: 2.70 (±0.97)	RT: >0.05 C: >0.05 RT: <0.05 * C: >0.05	RT/C: 0.34 RT/C: 0.28
Sukala et al., 2012 [20]	Adiponectin	RT: 5.6 (±1.9) AET: 6.7 (±3.3)	RT: 5.6 (±2.2) AET: 6.7 (±3.2)	RT/AET: >0.05	RT/AET: <0.001

Abbreviations (ordered alphabetically): AET: aerobic training; C: control; RBP4: Retinol-Binding Protein 4; RT: resistance training. * Significant difference (*p* ≤ 0.05). ^¶^: all studies included participants with body mass index ≥ 25.0 kg/m^−2^, except in the Kang et al. study (i.e., 23.4 kg/m^−2^).

**Table 4:** Summary of training protocols that reported significant improvements for specific adipokines.

	Aerobic Training				Resistance Training				
	Sessions per Week/Total Weeks	Intensity	Duration	Combined	Intensity	Duration	Sets	Repetitions	Exercises
Leptin, adiponectin and resistin	2/52	70–80% VO2max	40 min	*yes*	80% 1RM	20 min	--	--	Upper limb pull, horizontal push, knee extension, trunk flexion
Apelin, vaspin and visfatin	4/24	60–75% HRmax	60 min	*yes*	60–80% 1RM	60 min	2–3	8–10	Seated leg press, knee extension, knee flexion, chest press, lat pulldown, overhead press, bicep curl, tricep extension
RBP4	3/12	--	--	*--*	Circuit 55% HRR	--	3	8	Stair climbing, stationary cycling, resistance exercises (lat pull-down, abdominal, leg curl, leg extension, bicep curl)
Chemerin	3/12	50–70% VO2max	30 min	*yes*	50% 1RM	--	3	20	--
Omentin	3/12	55% HRmax	10–12 min	*yes*	50–55% 1RM	--	1–2	8–10	Leg extension, prone leg curl, abdominal crunch, biceps, triceps, seated calf raise

Abbreviations (ordered alphabetically): HRmax: maximum heart rate; HRR: heart rate reserve; RBP4: Retinol-Binding Protein 4; 1RM: one repetition maximum; VO2max: maximal oxygen uptake. “--” = Information was not specified in the study.

## Data Availability

Protocol is available upon reasonable request.
